# Interacting Effects of Instructions and Presentation Rate on Visual Statistical Learning

**DOI:** 10.3389/fpsyg.2015.01806

**Published:** 2015-11-30

**Authors:** Julie Bertels, Arnaud Destrebecqz, Ana Franco

**Affiliations:** ^1^Center for Research in Cognition and Neurosciences (CRCN), ULB Neuroscience Institute (UNI), Université Libre de Bruxelles (ULB)Brussels, Belgium; ^2^Fonds de la Recherche Scientifique – FNRSBrussels, Belgium

**Keywords:** visual statistical learning, implicit learning, explicit knowledge, conscious awareness, subjective measures

## Abstract

The statistical regularities of a sequence of visual shapes can be learned incidentally. Arciuli et al. (2014) recently argued that intentional instructions only improve learning at slow presentation rates as they favor the use of explicit strategies. The aim of the present study was (1) to test this assumption directly by investigating how instructions (incidental vs. intentional) and presentation rate (fast vs. slow) affect the acquisition of knowledge and (2) to examine how these factors influence the conscious vs. unconscious nature of the knowledge acquired. To this aim, we exposed participants to four triplets of shapes, presented sequentially in a pseudo-random order, and assessed their degree of learning in a subsequent completion task that integrated confidence judgments. Supporting Arciuli et al.’s (2014) claim, participant performance only benefited from intentional instructions at slow presentation rates. Moreover, informing participants beforehand about the existence of statistical regularities increased their explicit knowledge of the sequences, an effect that was not modulated by presentation speed. These results support that, although visual statistical learning can take place incidentally and, to some extent, outside conscious awareness, factors such as presentation rate and prior knowledge can boost learning of these regularities, presumably by favoring the acquisition of explicit knowledge.

## Introduction

Statistical learning refers to the ability to extract the regularities present in the environment. This process is essential, given the richness and complexity of our sensorial world. In particular, the learning of transitional probabilities is crucial in predicting forthcoming events on the basis of previous ones, an ability that has been documented in the auditory and visual modalities (see Krogh et al., 2013, for a review).

Many studies have convincingly demonstrated that statistical learning occurs incidentally and spontaneously, namely without any conscious attempt to extract the underlying structure of the material. In fact, when asked to verbally report on what they have learned, participants hardly manage to verbalize the acquired knowledge. These observations have led some authors to consider statistical learning as a form of implicit learning (Perruchet and Pacton, 2006).

However, at least two sets of evidence challenge the idea that statistical learning is an implicit process. First, recent studies show that most of the knowledge acquired through statistical learning is explicit. Participants are indeed aware of the fact that they learned regularities between elements (Franco et al., 2011; Bertels et al., 2012, 2013, 2015; Batterink et al., 2015). Second, several studies show that statistical learning benefits from intentionally searching for the regularities (Hamrick and Rebuschat, 2012; Kachergis et al., 2014; Stevens et al., 2014). Participants instructed to identify the structure of the material acquire more knowledge of the regularities than participants who learned them incidentally. Using explicit strategies to extract the structure of the material thus improves learning of this structure.

The extent to which intentional instructions influence statistical learning may depend, however, on the presentation rate of the stimuli during exposure. As a case in point, in a recent visual statistical learning (VSL) study, Arciuli et al. (2014) reported no effect of intentional instructions on the amount of knowledge acquired, and suggested that this absence of effect might be related to the fast pace of the stimuli. Similarly, Batterink et al. (2015), who used a comparable presentation speed, reported no impact of the intentional instructions given to the participants on their ability to learn words from an artificial language.

Arciuli et al. (2014) familiarized participants with twelve cartoon-like figures that were used to make up four triplets, namely groups of three figures presented sequentially, one at a time for 200 ms, in the order defined by the triplet to which it belonged. Crucially, triplets could not be segmented based on any spatial or temporal cues since the 200-ms inter-stimulus interval (ISI) was constant within and across triplets. Triplets were only identified by transition probabilities that were higher within a triplet than between triplets. Participants were either naïve concerning these regularities, or they were informed that the figures were presented by groups of three and that they would be later asked to identify them. The learning of the triplets was assessed using a two-alternative forced choice (2-AFC) recognition task. Although both groups performed above chance in this task, performance did not differ between the incidental and intentional learning groups. Arciuli et al. (2014) argued that the fast presentation rate in their study may have hindered the use of explicit strategies set up by the participants who were instructed to look for the regularities. Coherently, using the same paradigm with a slower presentation rate, Stevens et al. (2014) observed that participants in the intentional group outperformed those in the incidental group in a 2-AFC task.

Apart from possibly influencing the amount of the acquired knowledge, intentional instructions would also affect the extent to which knowledge of these regularities is available to consciousness. This issue has been seldom addressed in statistical learning studies. In a cross-situational statistical learning paradigm studying word–object associations, Hamrick and Rebuschat (2012) assessed the effects of learning conditions on participant awareness. These authors combined objective and subjective confidence measures (see also Poepsel and Weiss, 2014) and observed that participants who intentionally learned the associations mostly learned explicitly, while incidental participants were largely unaware that they learned any regularity.

In the present study, we put Arciuli et al. (2014) assumption to the test and measured whether the influence of intentional instructions depends on the presentation rate during exposure. Moreover, we also explored how instructions and presentation rate influenced the conscious vs. unconscious nature of the knowledge acquired; an issue that was not addressed in Arciuli et al.’s (2014) paper. To this aim, we used a visual sequential statistical learning paradigm, similar to the one we used in previous studies (Bertels et al., 2012, 2013, 2015) and mostly similar to Arciuli et al.’s (2014) paradigm, but in which we also used confidence judgments. Participants were first exposed to a stream of abstract visual shapes made up of the repeated presentation of four triplets and then tested on their knowledge of these triplets using a 4-AFC completion task in which confidence in their responses were taken after each trial. Crucially, we manipulated the instructions before exposure: half of the participants were told about the presence of regularities in the stream, while the other half was naïve as to their existence. We also varied the presentation rate of the stimuli: for half of the participants, shapes were presented at a fast pace [200 ms, with a 200 ms ISI between each shape, as in Arciuli et al.’s (2014) study], while the other half of shapes were presented at a slow pace [800 ms, with a 200 ms ISI, as in Stevens et al.’s (2014) study]. This resulted in four experimental groups.

Evidence of learning the regularities between shapes has been found under incidental learning conditions and with a similarly short SOA (Turk-Browne et al., 2005; Arciuli and Simpson, 2011; Bertels et al., 2012; see Conway and Christiansen, 2009 with even shorter SOA). Participants should then learn the triplets in all groups; namely, all groups should perform above chance level in the completion task. Intentional instructions should improve performance compared to incidental instructions (Hamrick and Rebuschat, 2012; Kachergis et al., 2014; Stevens et al., 2014). Importantly, this should only be the case with long SOA as, according to Arciuli et al.’s (2014) suggestion, a fast presentation rate may prevent the use of explicit strategies. It is indeed reasonable to think that time is needed between any two items in order to search for and check explicit hypotheses concerning the regularities between the shapes.

Under incidental learning conditions, participants in the slow-paced group should outperform those in the fast-paced group, as it has been shown that VSL improves when stimuli are presented slowly (Turk-Browne et al., 2005; Conway and Christiansen, 2009; Arciuli and Simpson, 2011; Emberson et al., 2011).

We also expected to observe both implicit and explicit knowledge acquisition under all experimental conditions. Indeed, previous VSL experiments have repeatedly shown that participants incidentally presented with a structured sequence of visual shapes at a fast presentation rate end up with a mix of both implicit and explicit knowledge (e.g., Bertels et al., 2012). Such a pattern of results has also been observed in participants who intentionally learned regularities between a word and an object with these stimuli presented at a slow pace (Hamrick and Rebuschat, 2012). Nevertheless, we predicted that the involvement of explicit strategies (i.e., in the intentional instructions group) would favor the acquisition of explicit knowledge of the regularities. We should thus observe more evidence of explicit knowledge under intentional than under incidental learning conditions, as in Hamrick and Rebuschat’s (2012) study (see also Guo et al., 2011 and Poepsel and Weiss, 2014).

Finally, we predicted that the amount of explicit knowledge would interact with the presentation rate in our VSL experiment. More explicit knowledge should be gained at a slow vs. fast presentation rate. Indeed, in a serial reaction time task experiment, Destrebecqz and Cleeremans (2001) showed that slowing the pace of the task improves explicit sequence knowledge acquisition. They reasoned that a slow rate offers more opportunities to implement explicit research strategies and to develop and link high-quality memory traces.

## Materials and Methods

### Participants

Participants were 130 students of the Université Libre de Bruxelles (104 women), ranging from 17 to 48 years (mean: 19.66). They received course credits for their participation. All reported (corrected-to-) normal vision. Participants were randomly assigned to one of four experimental conditions (Incidental *vs*. Intentional instructions and Fast vs. Slow pace, see below). They were tested up to three at a time in separate experimental booths.

The data from nine participants were discarded from the analyses: two because they did not perform the cover task conscientiously (one did not detect a single letter, the other detected 64 letters, although there were only 30 target letters in the stream), six because their average performance on the four-alternative forced choice (hereafter, 4AFC) task was less (*n* = 1) or more (*n* = 5) than two standard deviations above the overall average performance in their experimental group, and one because she felt dizzy during the experiment. The final sample was then made up of 121 participants, 59 in the Incidental instructions group (34 in the Fast and 25 in the Slow pace condition) and 62 in the Intentional instructions group (30 in the Fast and 32 in the Slow pace condition).

This study was carried out in accordance with the recommendations of the Ethics Committee of the Psychological and Educational Sciences Faculty at the Université Libre de Bruxelles. All subjects gave written informed consent in accordance with the Declaration of Helsinki. Participants were informed that they could withdraw from the experiment at any point.

### Stimuli

**FIGURE 1:**
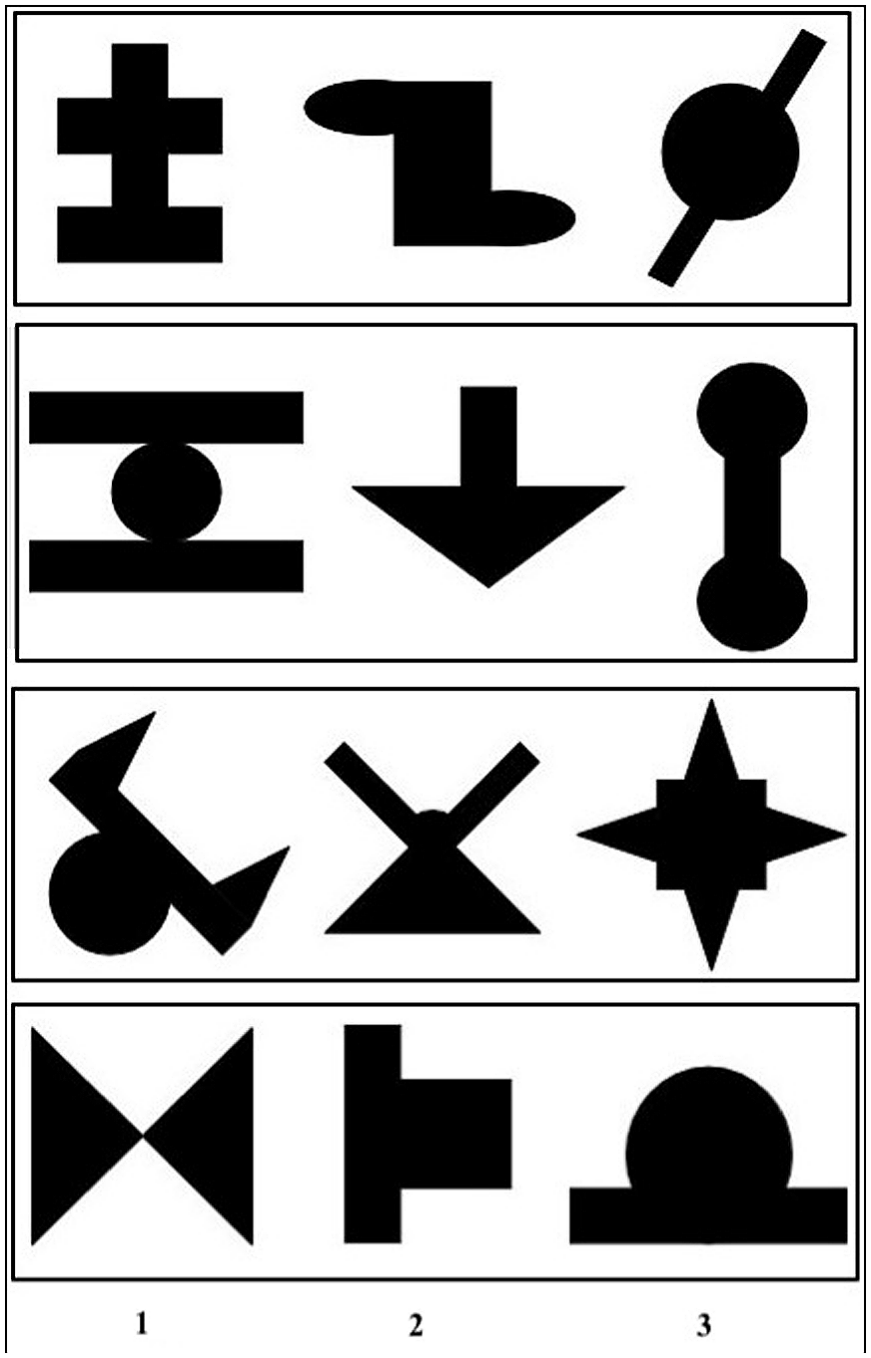
**Groups of three shapes constituting each of the four triplets, by order of presentation (1, 2, 3)**.

The visual stimuli consisted of 12 black shapes presented on a white background, adapted from Fiser and Aslin (2001). Each stimulus was about 3 cm by 3 cm. Stimuli constituted four ‘triplets’; namely, four sequences of three stimuli presented in a fixed order (**Figure 1**). As we did not report any effect of stimulus make-up in a previous study (Bertels et al., 2012), we only used one arrangement of four triplets (see also Arciuli and Simpson, 2011, 2012; Bertels et al., 2013, 2015).

### Apparatus

Stimulus presentation, timing and data collection were controlled using the Psyscope USB button box and Psyscope X B57 software (Cohen et al., 1993; Bonatti, 2010) running on a Mac mini.

### Procedure

#### Exposure

Exposure consisted of 1230 trials consisting of 100 repetitions of each of the 12 shapes constituting the triplets, plus 30 trials consisting of three presentations of 10 black letters shown on a white background. Stimuli were presented one at a time, for 200 ms in the Fast pace condition and for 800 ms in the Slow pace condition, with a 200 ms ISI in both conditions, resulting in an exposure phase of about 8 min in the Fast pace condition and of about 20 min in the Slow pace condition. Each of the twelve shapes was presented in the fixed order defined by the triplet to which it belonged. Triplets were pseudo-randomly presented: a given triplet was never presented twice in a row. The presentation of the shapes was randomly interspersed with the presentation of the letters. As a cover task, participants were asked to detect the letters by pressing a key. These data were not considered in the analyses, but to ensure that participants were motivated and attended the task, we discarded those who did not correctly perform the letter detection task (see above).

In the Incidental instructions group, participants were not told about the presence of regularities in the sequence. In the Intentional instructions group, participants were told that shapes were presented in groups of three, and that they will be asked to identify these sequences afterward. The exposure phase was followed by a 4AFC completion task.

#### Completion Task

The test consisted of a 4AFC task in which a triplet with one missing shape was presented on each trial. The triplet was first presented one shape at a time at the same rate as during the exposure phase, with a question mark in place of the missing shape. Then, the three shapes (including the question mark) were displayed side by side at the top of the screen, in the order defined by the triplet (see **Figure 1**). Participants had to pick one shape among the four presented to complete the triplet. These shapes were part of the triplets presented before, and their position matched with the position of the missing shape in the to-be-completed triplet. Participants answered by pressing one of four keys. There was no time constraint.

Each triplet was presented six times, resulting in 24 test trials. The same missing shape in the first, second or third position was thus presented twice, with a different presentation order of the four possible shapes. The sequence of completion trials was randomly presented across participants.

After each trial, participants expressed a binary confidence judgment about their completion response. They indicated whether they had guessed (i.e., they had no idea whatsoever concerning the correct response, they had answered at random) or remembered (i.e., they felt that their response was based on some recall of the learning material, even if they were not sure at all) by pressing one of two keys (for similar labels, see Dienes and Seth, 2010; Bertels et al., 2012, 2013, 2015).

## Results

### Overall Analyses

**Table 1** displays the average completion scores (i.e., the percentage of correct responses in the completion task) and confidence levels (i.e., the percentage of ‘Remember’ responses) for each experimental group.

**Table 1:** Average performance (i.e., percentage of correct responses) and confidence (i.e., percentage of ‘Remember’ responses) in the completion task by learning and presentation rate conditions, for all participants (*n* = 121).

	Performance			Confidence		
	Presentation rate			Presentation rate		
	Fast	Slow	Average	Fast	Slow	Average
Incidental learning	34.4 (3.2)	29.3 (3.7)	31.9 (2.5)	43.3 (4.6)	36.5 (5.4)	39.9 (3.6)
Intentional learning	34.3 (3.4)	47.8 (3.3)	41 (2.4)	43.1 (4.9)	56.3 (4.8)	49.7 (3.4)
Average	34.4 (2.3)	38.6 (2.5)		43.2 (3.4)	46.4 (3.6)	

*Standard errors are in parentheses.*

A univariate analysis of variance (ANOVA) was applied on the proportions of correct completion responses transformed by the arcsine function^1^, with Instructions (two levels: Incidental, Intentional) and Pace (two levels: Fast, Slow) as fixed factors.^2^ This analysis disclosed a significant effect of Instructions, *F*(1,117) = 6.887, *p* = 0.01, $\eta_{p}^{2}$ = 0.056. Participants performed better in the Intentional than in the Incidental group. The effect of Pace was not significant, *F*(1,117) = 1.883, *p* = 0.173. Nevertheless, the interaction between the two factors was significant, *F*(1,117) = 7.964, *p* = 0.006, $\eta_{p}^{2}$ = 0.064. A Welch’s ANOVA test^3^ revealed that the effect of Instructions was significant only when the presentation rate was slow, *F*(1,42.207) = 11.232, *p* = 0.002. Also, the effect of Pace was significant in the Intentional group, *F*(1,52.279) = 5.643, *p* = 0.021, but did not reach significance in the Incidental group, *F*(1,46.346) = 3.205, *p* = 0.08.

In a series of single-sample *t*-tests, we compared mean completion performance to the chance level (25%) in all groups and conditions.^4^ Mean completion performance was above chance in the Fast pace condition, in both Incidental and Intentional groups, *t*(33) = 5.107, *p* < 0.001, Cohen’s *d* = 1.778, and *t*(29) = 2.601, *p* = 0.014, Cohen’s *d* = 0.966, respectively. In the Slow pace condition, the mean completion performance differed significantly from chance in the Intentional group, *t*(31) = 4.785, *p* < 0.001, Cohen’s *d* = 1.719, but not in the Incidental group, *t*(24) = 1.853, *p* = 0.076.

Even though, participants performed above chance on average, about one third of them (*n* = 43) were actually at chance in the completion task, obtaining only 25% or less of correct responses. The proportion of participants at chance did not differ between Instruction groups (*n* = 21 in the Intentional and *n* = 22 in the Incidental group, χ^2^(1, *N* = 121) = 0.154, *p* = 0.695) or between presentation rate (*n* = 23 in the Fast and *n* = 20 in the Slow pace condition, χ^2^(1, *N* = 121) = 0.01, *p* = 0.922).

A univariate ANOVA applied on confidence levels (i.e., the proportion of ‘Remember’ responses) transformed by the arcsine function, with Instructions and Pace as fixed factors, disclosed a significant effect of Instructions, *F*(1,117) = 5.453, *p* = 0.021, $\eta_{p}^{2}$ = 0.045.^5^ Participants were more confident in the Intentional than in the Incidental group. The effect of Pace was not significant, *F* < 1. Nevertheless, the interaction between the two factors was significant, *F*(1,117) = 5.094, *p* = 0.026, $\eta_{p}^{2}$ = 0.042, and revealed that the effect of Instructions was significant only at the slow pace, *F*(1,54.526) = 9.195, *p* = 0.004.

In the following analyses, we focused on participants who performed above chance (*n* = 78) in order to investigate whether their knowledge was above the subjective criterion of consciousness. We explored whether they had some meta-knowledge about their statistical knowledge (for a similar procedure, see Bertels et al., 2012, 2013, 2015). To this aim, we used two indicators: the zero correlation and the guessing criteria (Dienes and Berry, 1997). The zero-correlation criterion is met when performance and confidence are not related to one another. In other words, if participants are not aware of their knowledge, high and low confidence ratings should be randomly assigned to correct and incorrect completions. Conversely, if performance is based on conscious knowledge, participants should be more confident in their correct responses than in their errors (Chan, 1992, unpublished doctoral thesis). According to the guessing criterion, knowledge is below the subjective threshold of consciousness when performance is above chance while participants claim to guess.

#### Participants Who Performed above Chance in the Completion Task (*n* = 78)

**Table 2** displays the average completion scores for each group (i.e., the percentage of correct responses in the completion task) and confidence levels (i.e., the percentage of ‘Remember’ responses) for participants who performed above chance in the completion task.

**Table 2:** Average performance (i.e., percentage of correct responses) and confidence (i.e., percentage of ‘Remember’ responses) in the completion task by learning and presentation rate conditions, for participants who performed above chance level in the completion task (*n* = 78).

	Performance			Confidence		
	Presentation rate			Presentation rate		
	Fast	Slow	Average	Fast	Slow	Average
Incidental learning	39.5 (3.4)	37.5 (4.4)	38.5 (2.8)	44.4 (5.7)	34.8 (7.3)	39.6 (4.6)
Intentional learning	45.6 (3.9)	58.9 (3.4)	52.2 (2.6)	48.6 (6.5)	62.5 (5.7)	55.6 (4.3)
Average	42.5 (2.6)	48.2 (2.8)		46.5 (4.3)	48.7 (4.6)	

*Standard errors are in parentheses.*

**FIGURE 2:**
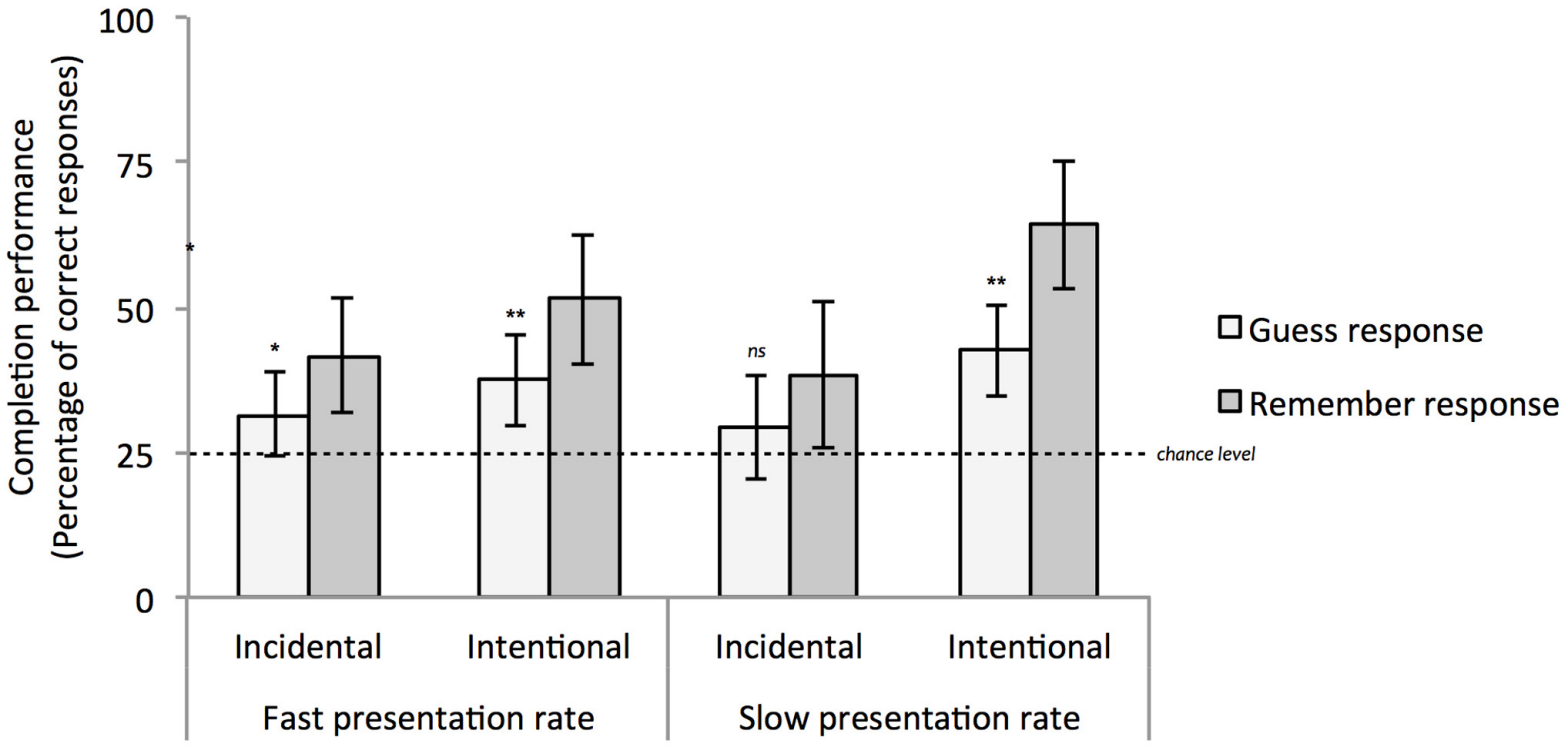
**Average completion performance for participants who performed above chance level, plotted separately by experimental group and for guess and remember responses.** Error bars represent 95% of confidence intervals around the means. ^∗^*p* < 0.05; ^∗∗^*p* < 0.01.

A repeated measures ANOVA was applied on the proportions of correct completion responses transformed by the arcsine function with Confidence (two levels: Guess, Remember) as a within-subject factor, and Instructions and Pace as between-subjects factors.^6^ Coherent with the previous analyses, this analysis revealed a significant effect of Instructions, *F*(1,64) = 14.394, *p* < 0.001, $\eta_{p}^{2}$ = 0.184: participants’ performance was higher in the Intentional than in the Incidental group (48.9% vs. 35.2%, respectively). The effect of Confidence also reached significance, *F*(1,64) = 9.974, *p* = 0.002, $\eta_{p}^{2}$ = 0.135: participants performed better when they reported remembering than when they claimed to have guessed (48.9% vs. 35.2%, respectively). No other main effect or interaction was significant, all *p* > 0.10. **Figure 2** displays mean performance by reported confidence separately for each experimental group.

Crucially, when participants claimed to have guessed, their performance significantly differed from chance level in the Intentional groups in both the Fast and Slow pace conditions, *t*(17) = 3.373, *p* = 0.004 and *t*(16) = 3.821, *p* = 0.002. For the Incidental group, although completion performance when reporting having guessed differed significantly from chance in the Fast pace condition, *t*(22) = 2.103, *p* = 0.047, this difference was not significant in the Slow pace condition, *t*(13) = 1.493, *p* = 0.159. Nevertheless, in the Incidental group, the difference between participants’ performance when they claimed to have guessed did not significantly differ as a function of Pace^7^, *F* < 1.

Taken together and according to the guessing criterion, such results suggest that completion performance in all experimental groups but one (the Incidental instructions/Slow pace group) was at least partly based on unconscious knowledge.

**FIGURE 3:**
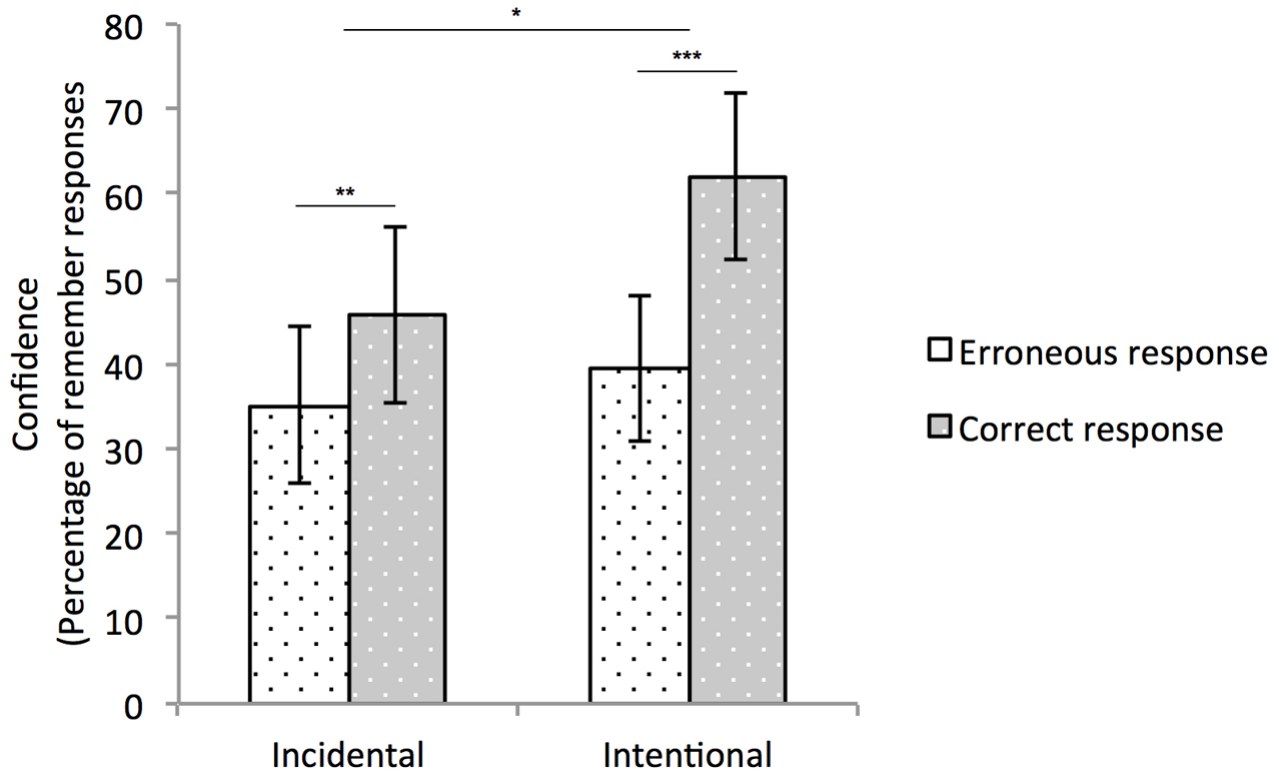
**Average confidence for participants who performed above chance level, plotted separately for each instructions group and for correct and incorrect completion responses.** Error bars represent 95% of confidence intervals around the means. ^∗^*p* < 0.05; ^∗∗^*p* < 0.01; ^∗∗∗^*p* < 0.001.

A repeated measures ANOVA applied on participants’ confidence transformed by the arcsine function with Completion response accuracy (two levels: Correct, Error) as a within-subject factor and Instructions and Pace as between-subjects factors revealed a significant effect of Response accuracy, *F*(1,74) = 20.511, *p* < 0.001, $\eta_{p}^{2}$ = 0.217. Overall, participants were more confident in their correct than in their incorrect completion responses (53.8% vs. 37.2%, respectively), indicating overall conscious knowledge by the zero-correlation criterion. The interaction between this factor and Instructions group was significant, *F*(1,74) = 4.447, *p* = 0.038, $\eta_{p}^{2}$ = 0.057: the difference between participants’ confidence for correct vs. incorrect completion responses was larger in the Intentional than in the Incidental group (22.7% vs. 10.6%). Both differences significantly differed from zero, indicating conscious knowledge by the zero-correlation criterion in both groups, *t*(40) = 5.006, *p* < 0.001 and *t*(36) = 3.385, *p* = 0.002. All other effects or interactions did not reach significance. **Figure 3** displays mean confidence by completion response accuracy plotted separately for each instructions group.

## Discussion

In the present study, we measured the effect of instructions to learn and of the pace of the task on learning the statistical regularities of a sequence of shapes. In a recent study, Arciuli et al. (2014) reported no difference between participants who were not informed about the presence of regularities in the stream (incidental instructions group) and participants who were informed and asked to extract these regularities (intentional instructions group). Interestingly, the authors suggested that the pace of the task might have hindered the search and the use of explicit strategies set up by intentional instructions to learn.

Here we aimed at directly testing this possibility by comparing the effect of intentional instructions to learn when visual stimuli are presented at a fast vs. slow presentation rate. We assessed the impact of instructions and pace on the amount of knowledge acquired through a forced-choice completion task and evaluated the conscious or unconscious nature of the acquired knowledge by the combined use of subjective confidence judgments.

Most participants learned the regularities since they performed above chance level in the completion task. Critically, as predicted by Arciuli et al. (2014), we observed that participants in the intentional group performed better than participants in the incidental learning group only when shapes were presented at a slow but not at a fast presentation rate. Some delay between sequence elements may be necessary for participants in the intentional learning group to set up explicit strategies in order to extract the statistical regularities. These strategies would consist of specifically attending to the successive order of shapes, trying to identify the regularities between them, and checking explicit hypotheses made regarding these regularities. It is also possible that intentional participants in the slow-paced group used the delay between shapes to attach verbal labels to them as it might be easier to remember a verbal auditory than a visual abstract sequence. However, this is pure speculation given that we did not ask participants about the strategies they set up in order to extract the regularities from the stream.

The fact that in the fast-paced group, intentional participants did not perform better than incidental participants does not mean that their statistical knowledge was the same. As a matter of fact, intentional participants knew that the sequence of shapes was not random, but the pace of the task was such that they could not use that knowledge to develop stronger representations of the sequential regularities.

Similarly, merely knowing that shapes were presented sequentially was not sufficient to improve confidence. Indeed, confidence judgments mirror performance results: intentional participants were more confident in their completion responses than incidental participants, but only when the pace of the exposure phase was slow. Confidence would thus depend more on the possibility to implement explicit strategies than on the mere knowledge that the sequence was regular.

While applying explicit strategies would be necessary for improving learning and confidence, merely knowing that regularities are present in the stream would be sufficient to influence the nature of the acquired knowledge. As a matter of fact, we observed that intentional instructions had an effect on the quality of the acquired knowledge, with regard to whether the presentation rate was fast or slow. Specifically, according to the zero-correlation criterion, learning was explicit in every experimental condition as performance was reliably related to confidence. Nevertheless, the magnitude of that relationship was stronger in intentional than incidental participants. Hence, knowing that shapes are sequentially presented favors the acquisition of conscious knowledge about them.

Learning, however, does not seem to be exclusively explicit. Rather, it consists of a mixture of explicit and implicit knowledge, as previously reported in the literature (e.g., Dienes et al., 1995; Bertels et al., 2012, 2013, 2015). As a matter of fact, participants performed above chance even when reporting to have had guessed. This is indicative of implicit knowledge according to the guessing criterion. Interestingly, participants acquired implicit knowledge of the sequences even under conditions that have been shown to maximize the acquisition of explicit knowledge; namely, under intentional learning conditions (see also Guo et al., 2011; Hamrick and Rebuschat, 2012). Moreover, in contrast with explicit knowledge, the amount of implicit knowledge did not vary as a function of instructions or presentation rate.

Surprisingly, we did not observe any effect of presentation rate under incidental learning conditions. Still, numerous studies have reported that visual sequential statistical learning benefits from slowing down the pace of stimulus presentation (Turk-Browne et al., 2005; Conway and Christiansen, 2009; Arciuli and Simpson, 2011; Emberson et al., 2011). Indeed, given the poor temporal resolution of visual processing, learning sequential regularities would be facilitated under less temporally demanding conditions (Emberson et al., 2011). Moreover, the additional exposure inherent to the slow presentation condition would boost learning of the regularities between shapes (Turk-Browne et al., 2005). Most probably, the absence of any effect of presentation rate in the incidental learning group in our study is related to the remarkably low average performance in the slow presentation rate condition. As a matter of fact, performance in that condition did not even significantly differ from chance level (although this was the case in the other conditions). Slow presentation of the shapes resulted in a 20-minute-long exposure phase. During exposure, participants were not involved in any task except the detection of scarcely presented letters. These conditions may have bored participants so that they did not use this extra time to process the stream more deeply. Previous studies reporting an effect of the presentation rate of the stimuli presented fewer occurrences of the shapes (hence leading to a shorter exposure phase, even in the slow presentation rate condition) and/or required participants to pay attention to the shapes to a greater extent than we did. In the present case, presenting shapes slowly without being instructed to look for regularities may have impaired the detection of these regularities since the likelihood that the to-be-associated shapes are simultaneously active in the short-term memory is relatively low (Frensch and Miner, 1994).

We should mention that, in the present study, we specifically investigated the effects of instructions and presentation rate on the acquisition of knowledge about order structure, but not ordinal structure^8^ (see Schuck et al., 2012, for evidence regarding the acquisition of these two forms of knowledge in sequence learning). While the former refers to triplet knowledge based on item-item associations (shape B comes after shape A in the triplet ABC), the latter refers to knowledge based on serial position-item associations (shape B comes in the second position). As a matter of fact, ordinal positional knowledge was not measured in our completion task as on each trial participants were presented with four alternatives that occurred in the same serial position in their respective triplet during exposure.

## Conclusion

The present results support Arciuli et al.’s (2014) claim that knowing the structure of a stream of visual shapes in advance only favors the learning of sequential regularities when participants have time to set up explicit strategies for extracting these regularities. We extended these findings by assessing how instructions impact the nature of the acquired order knowledge: We showed that even participants who were not informed about the regularities developed explicit knowledge of the sequences, but that those who were informed beforehand developed even more explicit knowledge. Critically, this was true at slow and fast paces, indicating that informing participants about the existence of regularities exerts a qualitative influence on the nature of the knowledge acquired.

## Author Contributions

JB, AD, and AF designed the experiment. JB and AF ran the experiment and analyzed the data. JB, AD, and AF wrote the article.

## Conflict of Interest Statement

The authors declare that the research was conducted in the absence of any commercial or financial relationships that could be construed as a potential conflict of interest.

## Footnotes

1 Since our distributions were not normally distributed and then violated the ANOVA’s assumption, we transformed our data using the arcsine function, as recommended by Howell (1997) for such a type of variable.

2 As suggested by a reviewer, we checked for any learning occurring during the task by considering the first and second parts of the test as a within-subject factor in a repeated measures ANOVA including Instructions and Pace as between-subject factors. Neither the main effect nor any interaction involving this factor were significant, all *p* > 0.20, suggesting that participants did not learn the triplets during the test.

3 Welch’s ANOVAs were run instead of one-way ANOVAs because Levene’s tests of homogeneity of variances highlighted unequal variances.

4 As indicated by Kolmogorov–Smirnov tests, three out of four of these variables were normally distributed. We therefore performed these single-sample *t*-tests on untransformed raw proportions.

5 As suggested by a reviewer, we also examined whether participants’ confidence improved between the first and second part of the test. A repeated measures ANOVA involving this factor as a within-subject factor did not reveal any significant effect nor any interaction involving this factor, all *p* > 0.06.

6 This analysis was run on 68 out of the 78 participants performing above chance since ten of them reported either to guess or to remember on every trial (so that we had no data for them for one of the level of the Confidence variable).

7 For similar reasons as stated above, single-sample *t*-tests were performed on raw proportions instead of transformed data.

8 We thank a reviewer for bringing this point to our attention.
